# Time Trends in the Incidence and Treatment of Extra-Abdominal and Abdominal Aggressive Fibromatosis: A Population-Based Study

**DOI:** 10.1245/s10434-015-4632-y

**Published:** 2015-06-05

**Authors:** Danique L. M. van Broekhoven, Dirk J. Grünhagen, Michael A. den Bakker, Thijs van Dalen, Cornelis Verhoef

**Affiliations:** Department of Surgical Oncology, Erasmus MC Cancer Institute, Rotterdam, The Netherlands; Department of Pathology, Maasstad ziekenhuis and Erasmus MC, Rotterdam, The Netherlands; Department of Surgery, Diakonessenhuis, Utrecht, The Netherlands

## Abstract

**Background:**

Aggressive fibromatosis (AF) is a locally infiltrating soft-tissue tumor. In a population-based study in the Netherlands, we evaluated time trends for the incidence and treatment of AF.

**Methods:**

In PALGA: Dutch Pathology Registry, all patients diagnosed between 1993 and 2013 as having extra-abdominal or abdominal wall aggressive fibromatosis were identified and available pathology data of the patients were evaluated. Epidemiological and treatment-related factors were analyzed with *χ*^2^and regression analysis.

**Results:**

During the study period, 1134 patients were identified. The incidence increased from 2.10 to 5.36 per million people per year. Median age at the time of diagnosis increased annually by B 0.285 *(P* = 0.001). Female gender prevailed and increased over time [annual odds ratio (OR) 1.022; *P* = 0.058]. All anatomic localizations, but in particular truncal tumors, became more frequent. During the study period diagnostic histological biopsies were performed more often (annual OR 1.096; *P* < 0.001). The proportion of patients who underwent surgical treatment decreased (annual OR 0.928; *P* < 0.001). When resection was preceded by biopsy, 49.8 % of the patients had R0-resection versus 30.7 % in patients without biopsy (*P* < 0.001).

**Conclusions:**

In this population-based study, an increasing incidence of extra-abdominal and abdominal-wall aggressive fibromatosis was observed. The workup of patients improved and a trend towards a nonsurgical treatment policy was observed.

Aggressive fibromatosis (AF; or desmoid-type fibromatosis) is a rare soft-tissue tumor that lacks the capacity to metastasize but may behave in a locally aggressive fashion. Knowledge on its epidemiology and etiology is limited. The Wingless/Wnt-pathway is involved although the mechanism is not fully understood.^1^–^3^ Three different subtypes are recognized as entities in the WHO-classification of desmoid-type fibromatose: extra-abdominal, abdominal, and intra-abdominal tumors.^4^ The first two mostly occur sporadic, whereas the latter has a correlation with familiar adenomatous polyposis (FAP).^5^

The incidence of AF was reported previously by Reitamo et al. in 1982, estimated at 2.4–4.3 per million people per year.^6^ Their studies on the etiology and epidemiology often are referred to in the current literature.^6^–^8^ The correlation of intra-abdominal AF with FAP has been subject of more recent studies.^9^–^11^ Current research on AF mainly focuses on treatment strategies. Surgery has until recently been the primary treatment modality. Data regarding the prognostic value of surgical margins and adjuvant radiotherapy is conflicting.^12^–^15^ New insights suggest that asymptomatic patients can be carefully watched without active treatment, and this is suggested by international (NCCN and ESMO) guidelines.^16^,^17^ Symptomatic patients with tumors that can be resected completely with acceptable morbidity should be offered surgery. In patients with symptomatic and “unresectable” disease, radiotherapy may be considered.^18^ Isolated limb perfusion can be considered for irresectable AF of the extremities.^19^ Systemic treatment also can be considered, although response rates are rather low.^20^–^22^

We evaluated time trends of the incidence and treatment of extra-abdominal and abdominal wall AF within the Dutch population.

## Methods

### Data Collection

The Dutch Pathology Registry PALGA was searched for patients with extra-abdominal or abdominal AF, whereas patients with intra-abdominal tumors were excluded.^23^ The epidemiology and treatment of intra-abdominal tumors are linked to FAP and are considered a different entity. Data on this entity in the Dutch population have been analyzed recently.^9^ The PALGA database contains encoded excerpts of all pathology examinations obtained by a diagnostic procedure, including tissue biopsy or resection since 1979 in selected laboratories and expanded to nationwide inclusion in 1991. The conclusion sections of all pathology reports were queried for available information concerning patient, tumor, and treatment characteristics. Age was categorized as <20, 20–44, 45–64, 65–79, and >80 years old. Tumor localization was categorized as head/neck, trunk (including breast, thoracic aperture and back), abdominal wall, extremity, and others. Reports were scored based on the encoding of procedures and details in the report as biopsy, resection or re-resection and on manifestation of the tumor (primary or recurrence). All patients undergoing re-resection were considered to have had a prior resection, even when pathology reports of the resection were missing. In case of patient records documenting recurrent disease, an attempt was made to retrieve details on the primary tumor. Due to incomplete data registration, patients with disease presentation before 1993 were excluded. The years of diagnoses were categorized as 1993–1998, 1999–2003, 2004–2008, and 2009–2013.

The primary objective was to analyze time trends in the incidence of AF. Trends of clinicopathological factors were analyzed as well as possible associations between the factors. The secondary objective was to analyze time trends in type of treatment, to which end the rate of resection was evaluated. Due to constrains in the pathology database structure, only data on pathology specimens, such as biopsy of resection were available. Information on other treatment strategies or outcome was not available.

In order to compare the patient cohort with the Dutch population, data from Statistics Netherlands were obtained. This is a registry for all general population data. We used information on demographics to calculate annual incidence rates and information on surgical treatments, hormonal drugs, and newborns to analyze possible etiological correlations.

### Statistical Analysis

Statistical analysis was performed using IBM SPSS Statistics 21. Continuous variables are shown as median and interquartile range (IQR), and categorical variables as numbers with percentages. Associations between clinicopathological variables were determined by *χ*^2^ analysis. Univariate logistic and linear regression analysis was performed to analyze trends over time. Results are shown as odds ratios (OR) or regression coefficient B (B) and with 95 % confidence intervals (CI). For all analyses, two-sided *P* < 0.050 was considered statistically significant.

## Results

A total of 1134 patients were diagnosed with extra-abdominal or abdominal wall AF between January 1993 and December 2013; there were 326 men and 808 women. Median age was 37 years [interquartile range (IQR) 30–50]. The distribution of demographic factors is shown in Table 1.

**Table 1:** Distribution of epidemiologic factors

	1993–1998		1999–2003		2004–2008		2009–2013	
	*N*	%	*N*	%	*N*	%	*N*	%
Gender								
Male	56	31.1	50	27.0	105	31.7	115	26.3
Female	124	68.9	135	73.0	226	68.3	323	73.7
Age (year)								
<20	18	10.0	14	7.6	29	8.8	39	8.9
20–44	115	63.9	124	67.0	170	51.4	239	54.6
45–64	37	20.6	33	17.8	85	25.7	112	25.6
65–79	10	5.6	11	5.9	39	11.8	43	9.8
80+	0	0	3	1.6	8	2.4	5	1.1
Localization								
Head/neck	14	8.0	13	7.1	20	6.1	27	6.2
Trunk	29	16.7	39	21.4	102	30.9	152	34.8
Abdominal wall	77	44.3	88	48.4	113	34.2	151	34.6
Extremity	45	25.9	32	17.6	68	20.6	85	19.5
Other	7	4.0	6	3.3	22	6.7	22	5.0
Unknown	2	1.1	4	2.2	5	1.5	0	0
Pathology reports								
Biopsy	13	7.2	39	21.1	69	20.8	130	29.7
Biopsy + resection	39	21.7	40	21.6	98	29.6	161	36.8
Resection	114	63.3	101	54.6	163	49.2	147	33.6
Unknown	14	7.8	5	2.7	1	0.3	0	0

In addition to the 1134 patients diagnosed as having AF, an uncertain diagnosis of AF was stated in the pathology excerpt in 213 patients. This latter group of patients did not change significantly over the years (*P* = 0.730). These patients were not included in the analyses for the present series.

### Epidemiologic Factors

**Fig. 1:**
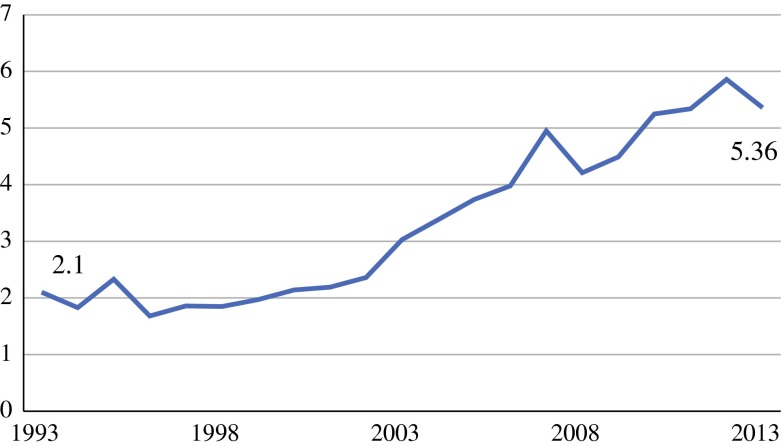
Incidence of aggressive fibromatosis, per million people

The incidence of extra-abdominal and abdominal wall AF increased over the study period, from 2.10 to 5.36 per one million people (*P* < 0.001; Fig. 1).

### Age

**Fig. 2:**
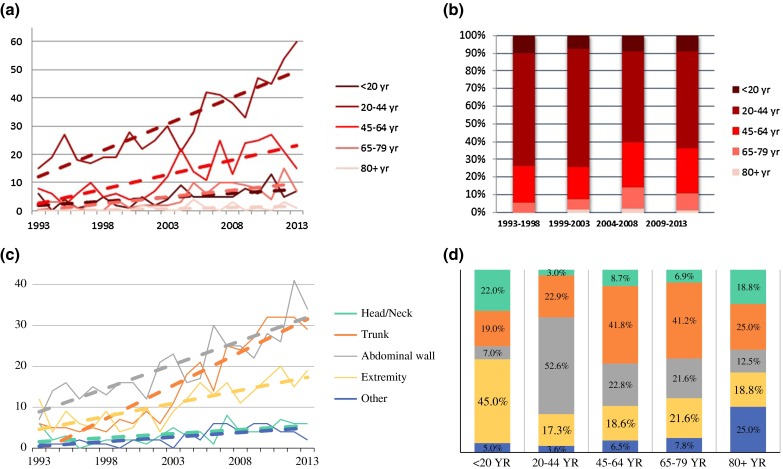
Distribution among age and localization. **a** Distribution among age during study period. **b** Percentage of age distribution. **c** Distribution among localization during study period. **d** Distribution of localization per age group

The median age increased annually by B 0.285 (95 % CI 0.114–0.455; *P* = 0.001). The median age in 1993–1998 was 34 years (range 27–45) and was 39 years (range 30–51) in 2009–2013. The absolute numbers increased in all age groups over time (Fig. 2a). However, the percentage of patients per age groups changed, mostly in patients aged 20–79 years (Fig. 2b). Analysis of the distribution among age groups showed a significant annual decrease in the percentage of patients aged 20–45 years (OR 0.977; 95 % CI 0.957–0.997; *P* = 0.027) and a trend towards an annual increase in the percentage of patients aged 45–65 years and 65–80 years (OR 1.017; 95 % CI 0.993–1.042; *P* = 0.173 and OR 1.035; 95 % CI 0.997–1.074; *P* = 0.069 respectively).

### Gender

The absolute numbers of both male and female patients increased over the years. The male–female ratio showed an increasing female predominance, ranging from 68.6 % in 1993–1998 to 73.6 % in 2009–2013.

### Anatomic Tumor Localization

Tumor localization was distributed as: 6.7 % head/neck, 29.0 % trunk, 38.6 % abdominal wall, 20.7 % extremity, and 5.1 % other (localization details were missing for 22 patients). Over the years, the absolute incidence in all groups increased (Fig. 2c). Analysis of the distribution of tumor localization showed a significant proportional increase in the percentage of patients with truncal localization (OR 1.057; 95 % CI 1.032–1.083; *P* < 0.001), whereas the percentage of patients with tumors in the abdominal wall decreased (OR 0.972; 95 % CI 0.952–0.993; *P* = 0.008).

### Associations Between Clinicopathological Factors

The distribution of tumor localization varied per age group (Fig. 2d). Extremity-based tumors were most common in patients younger than 20 years of age (45.0 %), whereas patients between 20 and 45 years most commonly harbored abdominal wall tumors (52.6 %); truncal tumors were predominantly seen in patients between 45 and 80 years of age (41.5 %). For patients older than 80 years of age, no dominant localization could be identified. The distribution of age groups and localization changed over the study period.

### Workup and Treatment

**Fig. 3:**
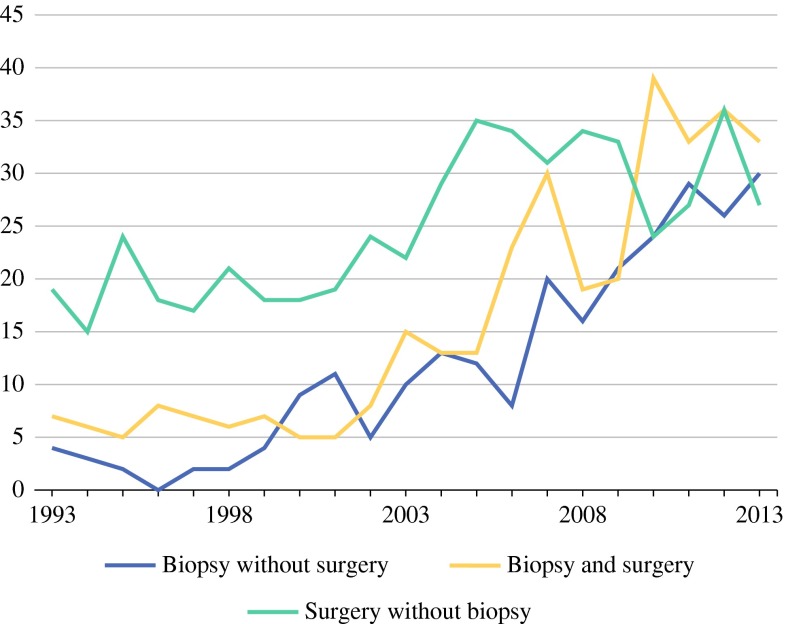
Type of pathology records per patient

In 251 patients (22.1 %) solely a biopsy report was retrieved; for 338 patients (29.8 %) a biopsy report and a pathology resection specimen report was retrieved, and for 525 patients (46.3 %) solely a pathology resection specimen report was retrieved. For 20 patients, the type of report was unknown (Fig. 3). From 1993–1998 to 2008–2013, the biopsy rate increased more than twofold: from 31.1 to 66.4 % (OR 1.096; 95 % CI 1.072–1.121, *P* < 0.001). The proportion of patients who underwent surgical resection decreased annually (OR 0.928; 95 % CI 0.902–0.954, *P* < 0.001). It was not known what treatment was offered to the patients who did not undergo surgery due to the nature of the database. Over time, surgical resection was increasingly preceded by biopsy. If a resection was preceded by biopsy, the resection margin status improved significantly (49.8 % R0-resection vs. 30.7 % in patients without biopsy; *P* < 0.001). Pathology reports did not discriminate between diagnostic or therapeutic resections. Median time between biopsy and resection was 1.6 months (IQR 0.9–2.7). The date of either biopsy or resection was missing for two patients. A substantial number of patients (210; 18.5 %) had a history of surgery in the same area where AF subsequently developed.

### Dutch Population

**Fig. 4:**
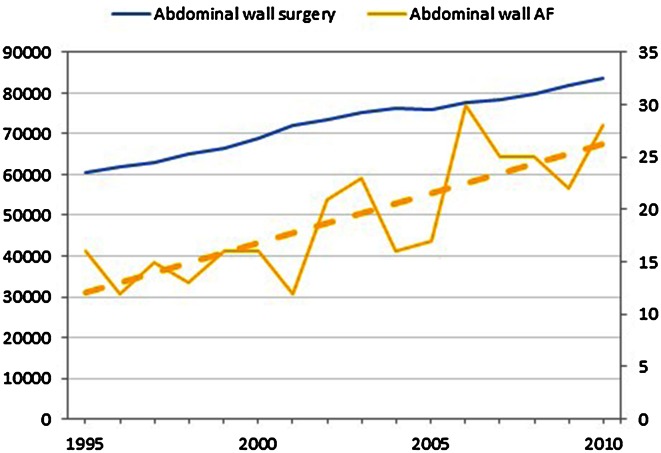
Absolute number of most common abdominal wall surgery, in relation to the absolute number of patients with abdominal wall AF (on secondary axis)

**Fig. 5:**
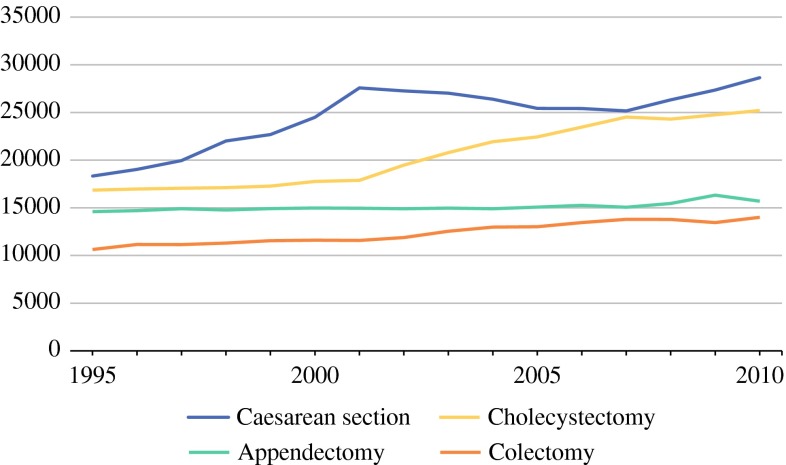
Abdominal surgery in the Netherlands

Since the abdominal wall was the most common tumor localization, we analyzed surgical trends in the Netherlands for the most common surgeries in this area (caesarean section, cholecystectomy, appendectomy, and colectomy).^24^ During the study period, surgical trauma to the abdominal wall increased (Figs. 4, 5). Due to minimal invasive techniques for many surgical interventions, the rate of laparotomy decreased and the rate of laparoscopic surgery increased.

Data on hormonal drugs was available for the period 2006–2012. During this period, the overall use of hormonal medication in the Netherlands remained stable.

The number of pregnancies of any gestational age was not available. The number of newborns per year was used as a surrogate, and during the study period this number decreased from 195.748 in 1993 to 171.341 in 2013.

## Discussion

The reference standard on the incidence and epidemiology of AF are Finnish studies by Reitamo et al.^6^–^8^ An incidence of 2.4–4.3 per million people was reported in those studies, using three methods of estimation (local, regional, and national). Distribution of disease was reported with a dominance of abdominal wall tumors (49 %) with variations per age groups. In the present population based study, a rising incidence of extra-abdominal and abdominal wall AF was observed from 2.1 to 5.36 per million people during the period 1993–2013. The distribution among age groups was similar to the Finnish studies, with a predominance of abdominal wall tumors in females aged 20–44 years. Remarkably, median age and female predominance increased over the years and the distribution of tumor localization shifted. The driving factor for these observed changes is unclear.

The PALGA database provided an elaborate overview of AF in the Netherlands. The nationwide coverage enabled epidemiological research on this rare disease. Then again, the available information was limited to the date and conclusion of the pathology reports. Although there was information on biopsy and resection, no information was available for nonsurgical treatments, which is a limitation of the present study. Still, important information could be extracted.

### Time Trends in Incidence

Explanations for the observed rising incidence of AF are not evident. If an increase in incidence occurs, this can be due to improved diagnostic modalities (i.e., for instance detection of previously unrecognized tumors by improved imaging, improved recognition of the disease by pathologists, or the start of a screening program) or due to a true increase in the incidence of the disease.

Improved registration and diagnostic tools are likely to have influenced the incidence figures to some extent. The changes in distribution of tumor localization might be an indication for a true change in disease. However, there are possible biases: other reasons could be an increased frequency of trunk computer-tomography scan or higher awareness due to screening programs.

Dutch guidelines on registration of neoplasms have changed over the years. The introduction of the third edition of the WHO Classification for Soft Tissue and Bone Tumours stimulated improvement of coding, enabling a better pathology registration.^25^ Due to the benign nature, this neoplasm is not registered among soft tissue tumors in the national cancer registries precluding verification of our data. The overall incidence of sarcomas has remained stable over the years at approximately 30–35 patients per million people, with a slight increase to around 40 patients per million people over the past 5 years.^26^

Knowledge on β-catenin and its application in the diagnostic setting around 2005 aided the pathologist in diagnosing AF with more confidence.^27^–^29^ Nevertheless, the percentage of uncertain diagnoses has not changed significantly over the years, indicating that some difficulty to distinguish AF from low-grade and reactive spindle cell proliferations remains. Awareness of the presence of AF and the realization of the importance of a correct diagnosis have improved. In addition, the association with FAP is better understood. Lastly, screening programs may have influenced the stage of diagnosis, such as the breast cancer screening program in asymptomatic people.

Documented etiological factors are surgical trauma, hormonal influences, and pregnancy.^6^–^8^ National data on these factors was obtained to provide some context for the study data. A hypothesis could be that the increased rate of surgical trauma would lead to an increase in AF. On the contrary, a limitation of surgical trauma by means of minimal invasive techniques could possibly decrease the risk of AF. The analyses of abdominal surgery and abdominal AF both showed increasing rates over the study period, which might be supportive of the first hypothesis.

The peak in occurrence of AF among fertile females is supportive of hormonal influences as an etiological factor. To test the hypothesis that a rise in hormonal levels would lead to an increase in AF, we compared data on hormonal drug use from Statistics Netherlands with the data from PALGA. Although the information on drug use was from a small period (2006–2012), the incidence of AF was rising during this period while the rate of hormonal drug use remained stable.

Pregnancy is seen as an etiological factor within the hormonal influences. Because no data on pregnancies in the patient cohort were available, we obtained the rate of newborns in the Netherlands during the study period. The rate of pregnancies of any gestational age was not available. The hypothesis that an increase in pregnancies (represented by the number of newborns) would lead to an increase in AF was not supported, as the rate of newborns was decreasing.

A more sensitive approach to test hormonal influences on AF, like analyzing hormonal receptors on the tumor, could provide more information but was not possible for the current study.

We would like to emphasize that the presented comparisons between data from PALGA and Statistics Netherlands are all based on hypotheses. Direct correlations for these etiological factors could not be explored and possible biases should be taken into consideration.

### Time Trends in Diagnosis and Treatment

Despite the aforementioned advances in diagnostic tools, the diagnosis of AF poses remaining challenges to the treating physicians. Although the rising incidence is most likely biased by diagnostic modalities and improved registration, the presented results showed an increasing number of patients being treated for AF.

The presented results suggest an improved workup procedure of patients as histological biopsies were more often obtained. Surgical resection following a biopsy diagnosis resulted in a significant higher rate of negative resection margins, underscoring the importance of the diagnostic process.

Treatment strategies changed in recent years and this is reflected in the present data. There has been a paradigm shift in the surgical treatment for AF patients. Before 2000, surgery with negative margins had been considered the standard of care for patients affected by AF, reflecting the same approach to extremity soft-tissue sarcomas. A reassessment has taken place by several groups, advocating a more conservative approach.^30^,^31^ The European consensus is currently set at an initial wait-and-see approach.^32^ The increasing number of patients undergoing nonsurgical treatment in the presented study indicated a tendency to adhere to this policy in the Netherlands. The growing knowledge and understanding of the etiology and involvement of CTNNB1-mutations will improve the diagnostic process.

During the past 25 years, developments in the available diagnostic modalities and changing treatment insights had an impact on the workup and treatment of extra-abdominal and abdominal wall AF. More insight in current epidemiologic trends and treatment-related trends was imperative. This population-based study reflected these changes and showed an overall incidence rise of AF. The reasons for the changing incidence, age distribution, and anatomic localization distribution remain to be further elucidated.
